# Perinatal N(G)-Nitro-L-arginine methyl ester administration decreases anxiety- and depression-like behaviors in adult mice

**DOI:** 10.31744/einstein_journal/2023AO0302

**Published:** 2023-11-23

**Authors:** Raoni Conceição Dos-Santos, Cláudio da Silva-Almeida, Bruno Guimarães Marinho, Rodrigo Rodrigues da Conceição, Wellington da Silva Côrtes, Ragab Gaber Ahmed, Roberto Laureano-Melo

**Affiliations:** 1 Universidade de São Paulo Faculdade de Medicina de Ribeirão Preto Department of Physiology Ribeirão Preto SP Brazil Department of Physiology, Faculdade de Medicina de Ribeirão Preto, Universidade de São Paulo, Ribeirão Preto, SP, Brazil.; 2 Universidade Estácio de Sá Rio de Janeiro RJ Brazil Universidade Estácio de Sá, Rio de Janeiro, RJ, Brazil.; 3 Universidade Federal Rural Rio de Janeiro Department of Physiological Sciences Seropédica RJ Brazil Department of Physiological Sciences, Universidade Federal Rural Rio de Janeiro, Seropédica, RJ, Brazil.; 4 Universidade Federal de São Paulo Laboratory of Endocrinology and Translational Medicine São Paulo SP Brazil Laboratory of Endocrinology and Translational Medicine, Universidade Federal de São Paulo, São Paulo, SP, Brazil.; 5 Beni-Suef University Faculty of Science Division of Anatomy and Embryology Beni-Suef Egypt Division of Anatomy and Embryology, Zoology Department, Faculty of Science, Beni-Suef University, Beni-Suef, Egypt.; 6 Universidade Barra Mansa Behavioral Physiopharmacology Laboratory Rio de Janeiro RJ Brazil Behavioral Physiopharmacology Laboratory, Universidade Barra Mansa, Rio de Janeiro, RJ, Brazil.

**Keywords:** Anxiety, Depression, NG-nitroarginine methyl ester, Nitric oxide, Pain perception, Mice

## Abstract

**Objective:**

We hypothesized that perinatal manipulations of the nitrergic system would affect adult animal behaviors.

**Methods:**

We tested this hypothesis by perinatally administering N(G)-Nitro-L-arginine methyl ester (L-NAME), a non-specific antagonist of nitric oxide synthase for 15 days and assessed anxiety- and depression-like behaviors in adult mice. At 70 days of age, the mice were subjected to a battery of tests consisting of the open-field, light/dark box, forced swim, and tail-flick tests. The tests were performed at two-day intervals, and the order of the tests within the battery was determined according to the progressive invasiveness degree.

**Results:**

L-NAME-treated animals exhibited decreased anxiety-like behavior in the light/dark box and open field tests, with no change in locomotor activity. Additionally, they demonstrated decreased depression-like behavior in the forced swim test and no change in pain perception in the tail-flick test.

**Conclusion:**

The nitrergic system is possibly involved in neural circuitry development that regulates behaviors since blocking perinatal nitric oxide production decreases anxiety- and depression-like behaviors in adult mice.

**Perinatal N(G)-Nitro-L-arginine methyl ester administration decreases anxiety- and depression-like behaviors in adult mice**

**Figure:**
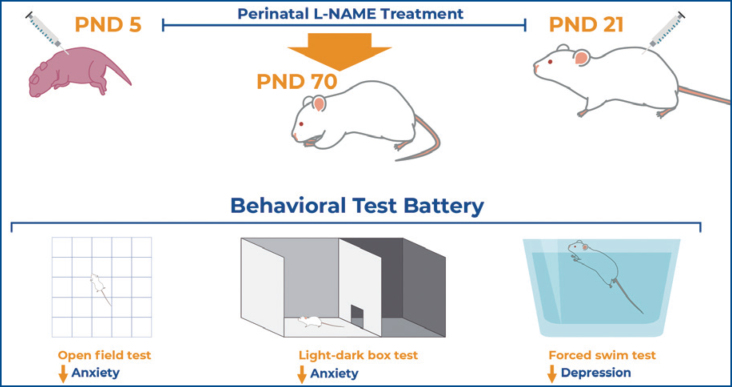


**Authors** Raoni Conceição Dos-Santos, Cláudio da Silva-Almeida, Bruno Guimarães Marinho, Rodrigo Rodrigues da Conceição, Wellington da Silva Côrtes, Ragab Gaber Ahmed, Roberto Laureano-Melo

**Correspondence** E-mail: rodriguescontato1@@hotmail.com

**DOI** DOI: 10.31744/einstein_journal/2023AO0302

**In Brief**

Dos-Santos et al. demonstrated that N(G)-Nitro-L-arginine methyl ester (L-NAME) administration, a nonspecific nitric oxide synthase antagonist, during the perinatal period promoted a decrease in anxiety- and depression-like behaviors in adult mice.

## INTRODUCTION

Anxiety and depression are mental illnesses that affect several individuals.^(1)^ When confronted with a potential threat, the risk assessment behavior involving fear is termed anxiety,^(2)^ which is divided into two types, state and trait anxiety. State anxiety refers to transiently increased anxiety, and trait anxiety refers to a disposition to experience anxiety in an otherwise harmless environment.^(3)^ Clinical studies have shown that depression and anxiety disorders are highly covalent,^(4)^ suggesting that their underlying mechanisms overlap. Animal models have been widely used to elucidate the mechanisms involved in mood and anxiety disorders, (termed anxiety- and depression-like behaviors).^(1)^ Thus, early life exposure to stress or environmental factors increases anxiety and depression later in life in other animals^(5)^ and humans.^(6)^

The nitrergic system has previously been shown to mediate the effects of stress on anxiety and depression.^(7)^ Nitric oxide (NO) is a gaseous modulator that influences several behaviors such as sleep, memory, learning, and reproductive behavior.^(8)^ Nitric oxide is synthesized via L-arginine oxidation by nitric oxide synthase (NOS).^(9)^N(G)-Nitro-L-arginine methyl ester (L-NAME) is a nonspecific NOS inhibitor. The results of L-NAME administration are controversial, including a reduction^(10-12)^ or elevation^(10-12)^ of anxiety-like behaviors in different experimental protocols. Additionally, L-NAME prevents anxiety and depression caused by acute or chronic stress^(13)^ and chronic morphine administration.^(14)^ Furthermore, NOS has demonstrated antidepressant effects,^(13,15,16)^ suggesting its participation in anxiety disorder and depression pathogenesis. Indeed, L-arginine, an NO precursor, counteracts the effects of L-NAME on anxiety,^(17,18)^ further supporting NO influence on anxiety.

Stress due to social isolation during adolescence increases trait anxiety in adult mice. L-NAME administration possibly reverses this effect, as the nitrergic system participates in anxiety and depression treatment.^(7)^ Exposure to several factors during the perinatal period can have long-lasting effects and contribute to the pathogenesis of various mood and anxiety disorders.^(19)^ Furthermore, we hypothesized that the chronic NOS blockade during early development would affect anxiety- and depression-like behaviors in adult mice. Additionally, L-arginine and L-NAME increases^(20)^ and decreases^(21,22)^ pain perception, respectively. These indicate that the endogenous nitrergic system is involved in nociception. However, the effects of chronic perinatal L-NAME administration on nociception remain unclear.

## OBJECTIVE

We hypothesized that perinatal manipulations of the nitrergic system would affect adult animal behaviors.

## METHODS

### Animals

Swiss Webster mice aged 60 days (approximately 35g), derived from the *Universidade Federal Rural do Rio de Janeiro* colony, were used in this study. After a 15 days acclimatization period, the mice were housed in plastic cages (30cm × 19cm × 13cm) and mated at a ratio of one female to one male. All animals used in this work were housed at a controlled temperature (20±2°C) with daily exposure to a 12 hours light/dark cycle and access to water and commercial rodent diet *ad libitum*. The environment of the mice was enriched using large/high cages and adequate nesting materials.

The litters after birth were adjusted to eight pups (four males and four females) per lactating mouse dam. Lactating dams and their offspring were separated into two groups (n=5). The Control Group consisted of dams whose offspring received 0.9% saline, whereas the treated one consisted of dams whose offspring received L-NAME hydrochloride (Sigma-Aldrich^®^, St. Louis, MO, USA) at a dose of 30mg/kg body weight from postnatal day (PND) 5 to 20, both via subcutaneous routes. This L-NAME dose was chosen because it is of its effectiveness in inducing anxiety-like behavior in adult mice.^(10,11)^ In each subject, a volume of 0.1mL per 10g of body weight was administered.

**Figure 1:**
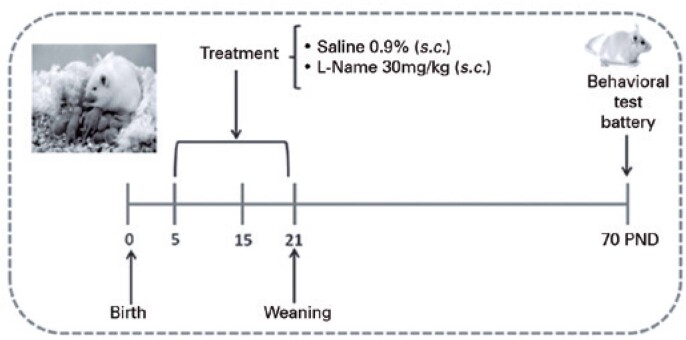
Representative timeline of the experiment

On PND 21, two male puppies of each litter were weaned to 10 animals per plastic cage (35cm × 50cm × 35cm). At 70 days of age, the mice were subjected to a battery of tests consisting of the open-field, light/dark box, forced swim, and tail-flick tests (Figure 1). The tests were performed at two-day intervals, and the order of the tests within the battery was determined according to the progressive invasiveness degree. All animals were anesthetized using an intraperitoneal injection of thiopental (90mg/kg) and euthanized by decapitation at the end of the experiment.

The experiments followed the Guide for the Care and Use of Laboratory Animals (NIH Publication Number 85–23, revised 1996) and were approved by the Institutional Animal Welfare Committee (protocol number: 23083.012282/2017). All efforts were made to minimize the number of animals used and refine the procedures.

### Open field

The effects of L-NAME programming on locomotor activity were assessed via an open-field test. Here, each animal was placed in the center of an acrylic box (80 × 80 × 30cm, evenly divided into 25 quadrants) and allowed to explore for 5 minutes, during which the number of quadrants traveled, number of rearings, entries into the central quadrant, central ratio, time spent in the central quadrant, and fecal boli were assessed. The number of quadrants traveled (distance traveled) and the number of rearings determined the locomotor activity.^(23)^ However, the time spent in the central area and the center/periphery ratio determined the anxiety-like behavior.^(24)^

### Light/dark box

The animals were individually placed on the dark side of a cage (45cm × 27cm × 27cm) that possessed two areas: a mildly lit (200 LUX) light side and a closed dark side. The mice were allowed to freely move between both sides for 5 minutes, and the time spent on the light side, number of transitions, and latency to enter the light side were assessed. This test is based on the aversion of rodents to lit and novel environments.^(25)^

### Forced swim

Mice were individually placed in a 25cm width cylinder, filled with 25°C water at a depth of 50cm to prevent them from escaping or touching the bottom of the tank.^(26)^ Rodents develop an immobile posture when confronted with inescapable and stressful situations.^(27)^ The animals were observed for 5 minutes, during which the immobility time and latency to the first immobility episode were assessed. These tests are related to low resilience, suggesting increased depression-like behavior.^(28)^ Furthermore, the climbing time was assessed.

### Tail flick

The tail-flick test was performed using a thermal pain threshold. The animal was gently restrained, and its tail was positioned over a tail-flick analgesia meter (Ugo Basile, Italy), in which high-intensity light was directed towards the tail. The light was automatically turned off when the animal moved its tail in response to a painful stimulus. Increased latency to tail flick indicates increased allodynia.^(29)^

### Statistical analysis

GraphPad Prism v7.0 was used for all statistical analyses. Outliers were identified using the ROUT test (Q = 10%) and removed from the analysis. For all comparisons, the Student's *t*-test was used. The effect sizes between the groups, which is the difference between means divided by the standard deviation (SD), were evaluated using Cohen's d analysis. In this measure, effect sizes were interpreted as small (0.2< d <0.5), moderate (0.5< d <0.8), and large (d >0.8). All data are presented as mean ± SD. Statistical significance was set at p<0.05.

## RESULTS

**Figure 2:**
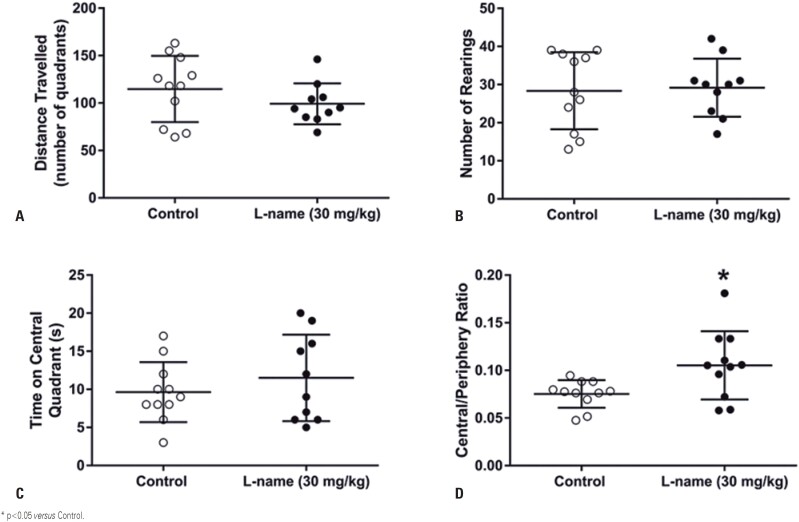
Perinatal L-NAME administration decreases anxiety-like behavior on the open field. The number of quadrants traveled (A) and the number of rearings (B) were not changed by L-NAME administration, which indicates that locomotor activity is unchanged. Time spent in the central quadrant (C) is not changed. However, the ratio of time spent in the central/periphery areas (D) indicates a decreased anxiety in the treated group

In the open-field test, the number of quadrants traveled (Figure 2A) and rearings (Figure 2B) remained unchanged. The time spent in the central quadrant did not change (Figure 2C). However, perinatal L-NAME administration significantly increased (Figure 2D; p=0.026) the ratio of the time spent in the center or periphery of the open field. These results indicated that L-NAME treatment decreased anxiety-like behavior with no changes in locomotor activity.

**Figure 3:**
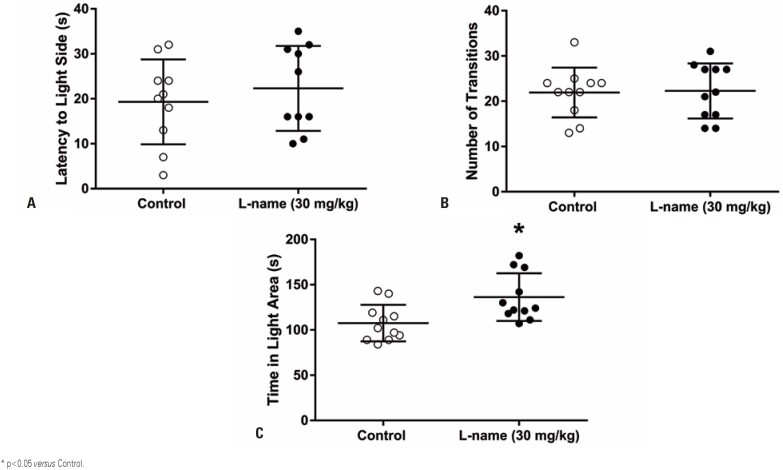
Perinatal L-NAME administration decreases anxiety-like behavior on the light-dark box. The latency to enter the light side (A) and the number of transitions (B) were unchanged. Time spent on the light area was increased in the animals (C), suggesting a decreased anxiety-like behavior

Similarly, perinatal L-NAME treatment increased the time spent on the light side of the light/dark box (Figure 3A; p=0.009) without changing the latency to enter the light area (Figure 3B) or the number of transitions between areas (Figure 3C). In parallel with the results of the open field test, decreased anxiety-like behavior was observed, with no changes in locomotor activity.

**Figure 4:**
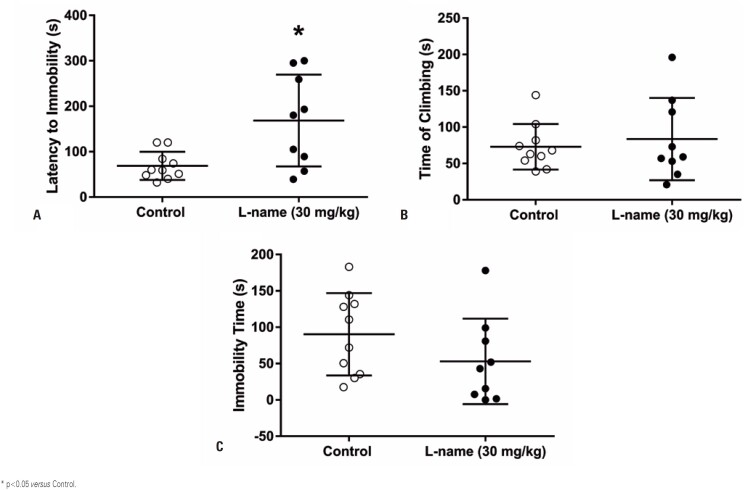
Perinatal L-NAME administration decreases depression-like behavior. The latency to immobility increases in the L-NAME treated mice (A), suggesting a decreased depression-like behavior. Time of climbing (B) and immobility time (C) were unchanged

**Figure 5:**
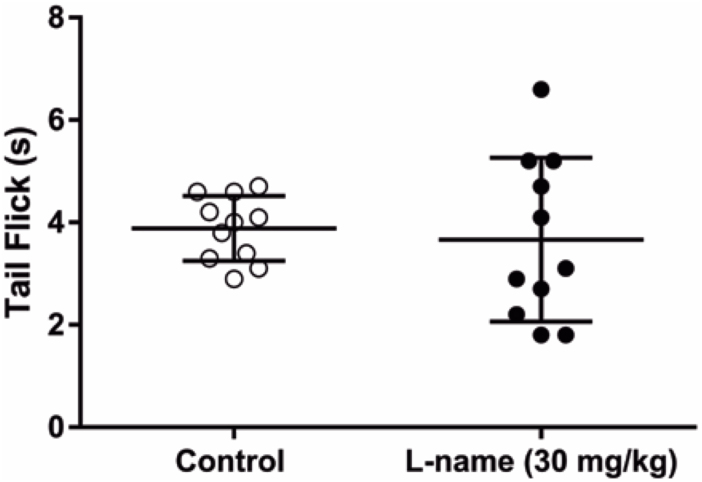
The tail-flick test showed no changes between groups. The nociception was not changed by the L-NAME

The forced swim test demonstrated that the latency to immobility increased in L-NAME-treated rats (Figure 4A; p=0.01). However, the time of climbing (Figure 4B) and immobility (Figure 4C) did not change. Thus, depression-like behavior decreased in L-NAME-treated mice. Regarding the pain threshold, the latency to tail flick did not change after L-NAME administration (Figure 5).

According to Cohen's d analysis, perinatal L-NAME treatment had a strong effect on the center ratio (d = 1.33), time on the light side (d = 1.22), latency to immobility (d = 1.33), and immobility time (d = 0.85%) (Table 1). A medium effect was observed for the number of quadrants traveled (d = 0.53).

**Table 1:** Numbers in italic represent moderate magnitude of the effect, whereas bold numbers represent large magnitude of the effect

Behavioral parameters		Cohen's *d*
Open Field Test	Total squares crossed	*0.53*
	Center ratio	**1.33**
	Time in center zone	0.38
	Rearing	0.09
Light-Dark Box Test	Latency to light	0.06
	Time in light side	**1.22**
	Transitions	0.31
Forced SwimTest	Latency to immobility	**1.33**
	Immobility time	**0.85**
	Climbing time	0.23
Tail Flick Test	Pain threshold	0.18

## DISCUSSION

Nitric oxide is widely expressed throughout the body and controls neurotransmission, inflammation, and the cardiovascular system.^(30)^ When synthesized, it readily crosses membranes and influences both the producing cell and neighboring cells.^(31)^ Nitric oxide is produced from L-arginine through NOS action, with these enzymes existing in three isoforms: neuronal, endothelial, and inducible.

Neuronal and endothelial NOS are constitutively calcium-dependent enzymes. Additionally, inducible NOS is expressed in certain tissues in response to immunological stimulation.^(32)^ All isoforms are present in the central nervous system,^(31)^ and endogenous NO is implicated in modulating the release of several neurotransmitters in distinct brain areas.^(33)^ The nitrergic system is critical in the developing brain. Postnatal administration of NOS antagonists reduces synapse formation in the hypothalamus, prefrontal cortex, and hippocampus,^(34)^ and the changes in synapse formation persist in the hippocampus of adult rats.^(35)^ Additionally, NOS blockade in the first week of life induces long-lasting effects on behavior, with decreased memory formation, but no effect on locomotion, as assessed in the open field test.^(36)^ Altogether, these observations indicate that NO induces synaptic plasticity in the brain until adulthood. These changes may contribute to increased susceptibility to anxiety and depression. Thus, L-NAME administration during critical developmental periods may prevent synapse formation. Herein, nitrergic transmission disruption caused by L-NAME administration during early life decreased anxiety- and depression-like behaviors in adults without affecting pain perception.

Nitric oxide is possibly involved in anxiety generation in adult rodents because blocking NO release induces anxiolytic effects^(37-39)^ and the arginine donors, thus increasing NO production and anxiety.^(40,41)^Additionally, NO antagonism potentiates the effects of antidepressant^(42,43)^ and anxiolytic^(41)^ drugs and attenuates stress-induced increase in anxiety and depression.^(13)^ L-arginine may blunt the effects of antidepressant drugs.^(42,43)^ Additionally, L-NAME reverses the increase in anxiety- and depression-like behaviors caused by social isolation stress. This effect was not induced by 7-nitroindazole, a specific neuronal NOS inhibitor, suggesting that it is not dependent on neuronal NOS.^(7)^ The interaction between the nitrergic system and anxiety- and depression-like behaviors is possibly dose-dependent since low L-arginine and L-NAME doses increased the antidepressant effect of scopolamine in the FST.^(44,45)^ Notably, the antidepressant effect of mecamylamine may be potentiated by the co-administration of low L-NAME or L-arginine doses. Indeed, L-NAME administration was more effective than L-arginine administration.^(46)^ The results of the light/dark box and open field tests indicated a decrease in anxiety-like behavior, with no changes in locomotor activity. Meanwhile, the forced swim test demonstrated that depression-like behavior decreased in the L-NAME-treated mice. Thus, disrupting NO production during development induces long-lasting changes in anxiety- and depression-like behaviors in mice. Nitric oxide-induced changes in the microglia and inflammatory cytokines in the brain mediate the effects of chronic stress on anxiety and depression^(45)^ and inducible NOS and not neuronal NOS is involved in anxiety and depression generation after chronic stress.^(7)^Increases in NO possibly mediate the formation of anxiety and depressive neurocircuitry in young mice. Based on this assumption, L-NAME administration during early development partially impedes this circuit formation. Consequently, adult animals are less prone to anxiety- and depression-like behaviors.

Nitric oxide is essential for processing nociceptive signals. Nitric oxide signaling sensitizes the spinal cord induc hyperalgesia.^(18)^ L-arginine increases^(20)^ and L-NAME decreases^(21,22)^ pain perception, respectively. We assessed whether perinatal L-NAME administration affected pain perception in adult mice. The results observed in these experiments were insignificant; thus, NO did not appear to be involved in pain perception development.

## CONCLUSION

The nitrergic system seems to be involved in developing the neural circuitry that regulates behavior since blocking nitric oxide production during early development decreases anxiety- and depression-like behaviors in adult mice. Therefore, the nitrergic system is a potential target for studying trait anxiety progression. Although nitric oxide participates in adult pain perception, the nitrergic system may not be involved in the nociception circuitry.
